# Progressive Loss of Acromioclavicular Joint Reduction Correlated with Progressive Clavicular Tunnel Widening after Coracoclavicular Stabilization in Acute High-Grade Acromioclavicular Joint Injury

**DOI:** 10.3390/jcm13154446

**Published:** 2024-07-29

**Authors:** Korakot Maliwankul, Pathawin Kanyakool, Prapakorn Klabklay, Wachiraphan Parinyakhup, Tanarat Boonriong, Chaiwat Chuaychoosakoon

**Affiliations:** Department of Orthopedics, Faculty of Medicine, Prince of Songkla University, Hat Yai, Songkhla 90110, Thailand

**Keywords:** acromioclavicular joint injury, clavicular tunnel widening, loss of reduction, radiography

## Abstract

**Objectives**: This study aimed to compare 24-month radiographic follow-ups of clavicular tunnel widenings (CTWs) and coracoclavicular distances (CCDs) and examine correlations between these measurements in patients following combined coracoclavicular stabilization and acromioclavicular capsule repair in treatment of acute high-grade acromioclavicular joint injury. **Methods**: This retrospective study reviewed the records of patients with acute Rockwood type V acromioclavicular joint injury who underwent surgery within 3 weeks after their injury. All patients had follow-ups at 3 and 6 months and 1 and 2 years. The CTWs were measured on anteroposterior radiographs between the medial and lateral borders at the superior, middle and inferior levels of the tunnels. On anteroposterior radiographs of both clavicles, the CCDs were measured at the shortest distance between the upper border of the coracoid process and the inferior border of the clavicle and reported as the CCD ratio, which was defined as the ratio of the affected and unaffected clavicles. At the final follow-ups, clinical outcomes were assessed using American Shoulder and Elbow Surgeons (ASES) scores. **Results**: This study included seventeen men and six women with a mean age of 47.26 ± 10.68 years. At the final follow-ups, the mean ASES score of all patients was 95.28 ± 3.62. We found a significant correlation between the increase in the CTWs and the increase in the CCD ratios (Spearman’s rho correlation coefficient range 0.578–0.647, all *p*-values < 0.001). **Conclusions**: We found long-term postoperative widening of the clavicular tunnels, which correlated positively with a gradual postoperative decline in the acromioclavicular joint alignment reductions.

## 1. Introduction

Acromioclavicular (AC) joint injuries account for 9–12% of shoulder girdle injuries [1,2]. The goal of treatment is to achieve anatomical reduction, stable fixation, and preinjury functional status. Surgical intervention is the recommended treatment, but there is a lack of general agreement on the best surgical technique [3,4,5]. Several techniques are commonly used for treatment of high-grade AC joint injuries, divided into AC stabilizing techniques (hook plate fixation, tension band wiring, AC capsule repair or reconstruction) and coracoclavicular (CC) stabilizing techniques (CC stabilization or reconstruction) [6,7]. In recent years, a variety of combined CC-AC stabilizing procedures have been developed for biomechanical improvement of both vertical and horizontal stabilities [7,8], which have become popular. However, several studies have reported complications following this combined procedure, including postoperative loss of reduction, clavicular tunnel widening, AC joint arthritis, distal clavicular osteolysis, and CC ossification [6,9,10]. 

The widening of clavicular tunnels and postoperative loss of reduction are the main undesirable radiological outcomes after CC stabilization [6,7,8]. Some studies have reported an effect of tunnel widening and radiographic loss of reduction in treating high-grade AC joint injury after CC stabilization [11,12,13]. These studies, however, had a follow-up time of only 1 year, which could have been too short for this particular type of complication to manifest.

The purpose of this study was to evaluate clinical and radiographic outcomes after combined CC stabilization and AC capsule repair in patients with acute high-grade AC joint injuries and correlations between the widening of clavicular tunnels, postoperative loss of reduction and clinical outcomes at 24-month follow-ups. Our hypothesis was that the widening of the clavicular tunnels would be correlated with postoperative loss of reduction and clinical outcomes.

## 2. Materials and Methods

This retrospective investigation received ethical approval from the Ethics Committee of our faculty. The requirement for informed consent from the study subjects was waived by the IRB due to the retrospective study design (REC 64-401-11-3). All procedures were performed in accordance with the relevant guidelines and regulations.

We retrospectively reviewed, between September 2021 and November 2021, the records of patients who underwent combined CC stabilization and AC capsule repair between January 2012 and December 2019. The study included patients with acute Rockwood type V AC joint injury who underwent surgery within 3 weeks after the injury and had a minimum of 2 years of complete clinical and radiographic follow-ups. Patients with a history of fracture or any serious injury around the shoulder girdle or prior shoulder surgery were excluded from the study. A total of 23 patients who met the inclusion criteria were identified from the hospital records. The demographic and clinical data were recorded on a standard form including age, sex, mechanism of injury, side of injury, time between injury and surgery, and functional scores as assessed by the American Shoulder and Elbow Surgeons (ASES) tool. For the radiographic data, we measured the widths of the medial and lateral clavicular tunnels and coracoclavicular distance (CCD) at regular follow-up periods until the final 24-month postoperative follow-ups; other radiological outcomes were also recorded, including postoperative clavicular fractures, AC joint arthritis, distal clavicular osteolysis, and CC ossification.

### 2.1. Surgical Technique and Postoperative Care

**Figure 1:**
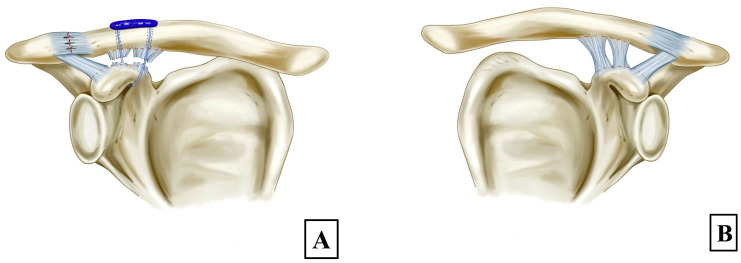
Illustrations showing a 50% decrease in the coracoclavicular (CC) distance on the affected side (**A**) compared to the unaffected side (**B**).

All combined CC stabilization and AC capsule repair procedures during the study period were performed by a single experienced orthopedic surgeon (P.K.). All surgeries were performed with the patient under general anesthesia in the beach chair position. A fluoroscope was prepared on the contralateral side to assess the reduction. A 5 cm saber incision was made from the distal clavicle to the coracoid process. An Ethibond No. 2 suture was used to make a loop under the coracoid base and behind the coracoacromial ligament from the medial side to the lateral side of the coracoid base to avoid the risk of iatrogenic neurovascular injury [14], then the suture was replaced with a shuttle loop using a shuttle relay technique. Next, the operation moved to the clavicular area, where the deltotrapezial fascia over the clavicle was incised along the longitudinal plane and the fascia was peeled off from the bone using a periosteal elevator. An Ethibond No. 5 suture was folded into a double length and the looped end passed under the coracoid base using a shuttle relay suture and then the same looped end was passed anterior to the anterior border of the clavicle. The open end of the doubled loop was passed posterior to the posterior border of the clavicle. The AC joint dislocation was then reduced and the suture was tightened to maintain the reduction. The reduction technique of decreasing the CCD to 50% of the unaffected side was used [7] (Figure 1A,B) and then the reduction was checked with the fluoroscope. Lateral and medial clavicular tunnels were created using a 2.5 mm drill bit at 2.5 cm and 4.5 cm from the AC joint [15], respectively. Two FiberWire No. 5 sutures (Arthrex) were passed under the coracoid base and the two same ends of the sutures passed through the clavicular tunnels and then a small plate and tightened. The final reduction was checked with the fluoroscope before all sutures were tightened. The AC capsule was repaired using Ethibond No. 2 sutures and the deltoid fiber was repaired using Vicryl No. 3–0. The skin was then closed layer by layer. 

All patients were advised to follow a structured rehabilitation timeline after their surgery. Immediately postoperatively, they were instructed to wear an arm sling and start pendulum exercises. This regimen continued for the first 6 weeks. At the 6-week mark, with the discontinuation of the sling, the patients began a progressive series of passive and active-assisted range-of-motion exercises. These included shoulder movements in forward flexion, abduction, and both internal and external rotations. Additionally, strengthening exercises using a resistance band were introduced. Following this phase, 3 months after the surgery, the patients were allowed to return to their full normal activities.

### 2.2. Clinical Evaluations

All patients were followed for at least 2 years, and they were evaluated with the ASES questionnaire at the last follow-up by an independent physician’s assistant. All postoperative complications from the hospital medical records including postoperative surgical wound infections, fractures, and reoperations were recorded for all patients. 

### 2.3. Radiographic Evaluations

**Figure 2:**
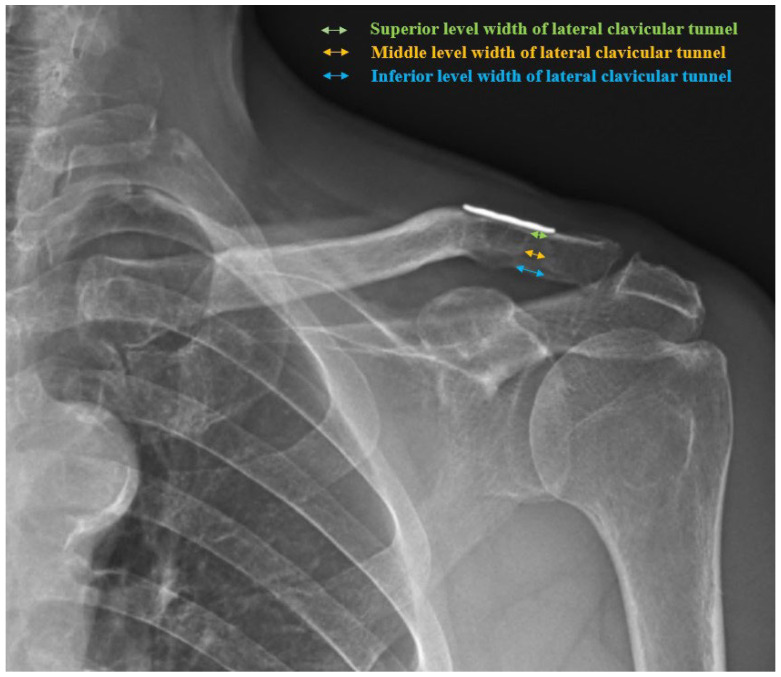
A 24-month follow-up AP radiograph of an affected shoulder showing widening of the medial and lateral clavicular tunnels after combined CC stabilization and AC capsule repair. The widths of the lateral clavicular tunnel were measured at the superior (green arrow), middle (orange arrow), and inferior (blue arrow) levels in millimeters (mm).

Anteroposterior (AP) images of the affected shoulder and both clavicles were taken immediately postoperatively and at 3-month, 6-month, 12-month, and 24-month follow-ups, and the widths of the medial and lateral clavicular tunnels were measured at the superior, middle, and inferior levels in millimeters (Figure 2), and these values were compared to assess the degrees of widening. 

**Figure 3:**
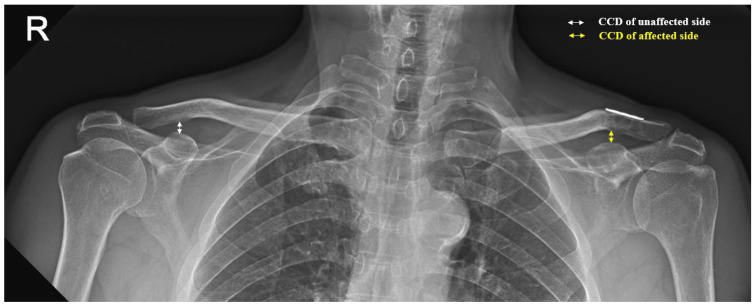
A 24-month postoperative AP radiograph of both clavicles showing the coracoclavicular distance (CCD), defined as the shortest distance between the uppermost level of the coracoid process and the lowest level of the clavicle. The CCD ratio was defined as the CCD of the affected (yellow line) and unaffected (white line) clavicles. In this case, the CCD ratio is CCD_affected_/CCD_unaffected_ = 1.01.

The AP images of both clavicles were used for measuring the CCD, which was defined as the shortest distance between the uppermost level of the coracoid process and the lowest level of the clavicle in millimeters. We assessed changes in the reductions using the CCD ratios between the affected and unaffected sides (Figure 3). We classified the reductions as adequate reduction or loss of reduction based on the CCD ratio. An adequate reduction was defined as a CCD ratio ≤ 1.1 and loss of reduction was defined as a CCD ratio > 1.1. The other radiological outcomes recorded were postoperative clavicular fracture, AC joint arthritis, distal clavicular osteolysis, and CC ossification.

### 2.4. Statistical Analysis

A comprehensive statistical analysis was conducted to evaluate the data collected in this study. To minimize the potential influence of measurement bias, each parameter was measured three times by two independent orthopedists and the average of all measurements was used to calculate the mean and standard deviations (SD) for all parameters at the immediate postoperative period and the regular follow-up points at 3 months, 6 months, 1 year, and 2 years.

The statistical analysis was performed using the R program and the “epicalc” package (version 4.1.1; R Foundation for Statistical Computing, Vienna, Austria). We used a linear mixed-effects model to rigorously analyze the continuous longitudinal data collected in our study. To explore the relationships between clavicular tunnel widths and changes in the CCD ratios, Spearman’s correlation coefficient was calculated. Subsequently, a power analysis was conducted to determine the sample size required to detect significant effects between the clavicular tunnel widths and CCD ratios. The differences in the widths of the medial and lateral clavicular tunnels at the superior, middle, and inferior levels were analyzed using Generalized Estimating Equations (GEE). The mean scores from the ASES evaluations were recorded with means ± standard deviations at the final 24-month follow-ups. These scores were compared between patients with or without CC ossification and with or without AC joint arthritis. Inter- and intra-observer reliabilities were calculated to assess the consistency and reproducibility of the measurements between the two observers and for the repeated assessments by the same observers. A significance threshold of *p*-value < 0.05 was used to indicate statistical significance.

## 3. Results

There were twenty-three patients (seventeen men and six women) included in this study with an average age of 47.26 ± 10.68 years. The causes of injury were traffic accident in eighteen patients and sports injury in five patients. The average time from injury to surgery was 7.08 ± 2.01 days. Twelve patients had right shoulder injuries, and eleven patients had left shoulder injuries, with one left-handed individual in each group.

### 3.1. Radiographic and Functional Outcomes

**Figure 4:**
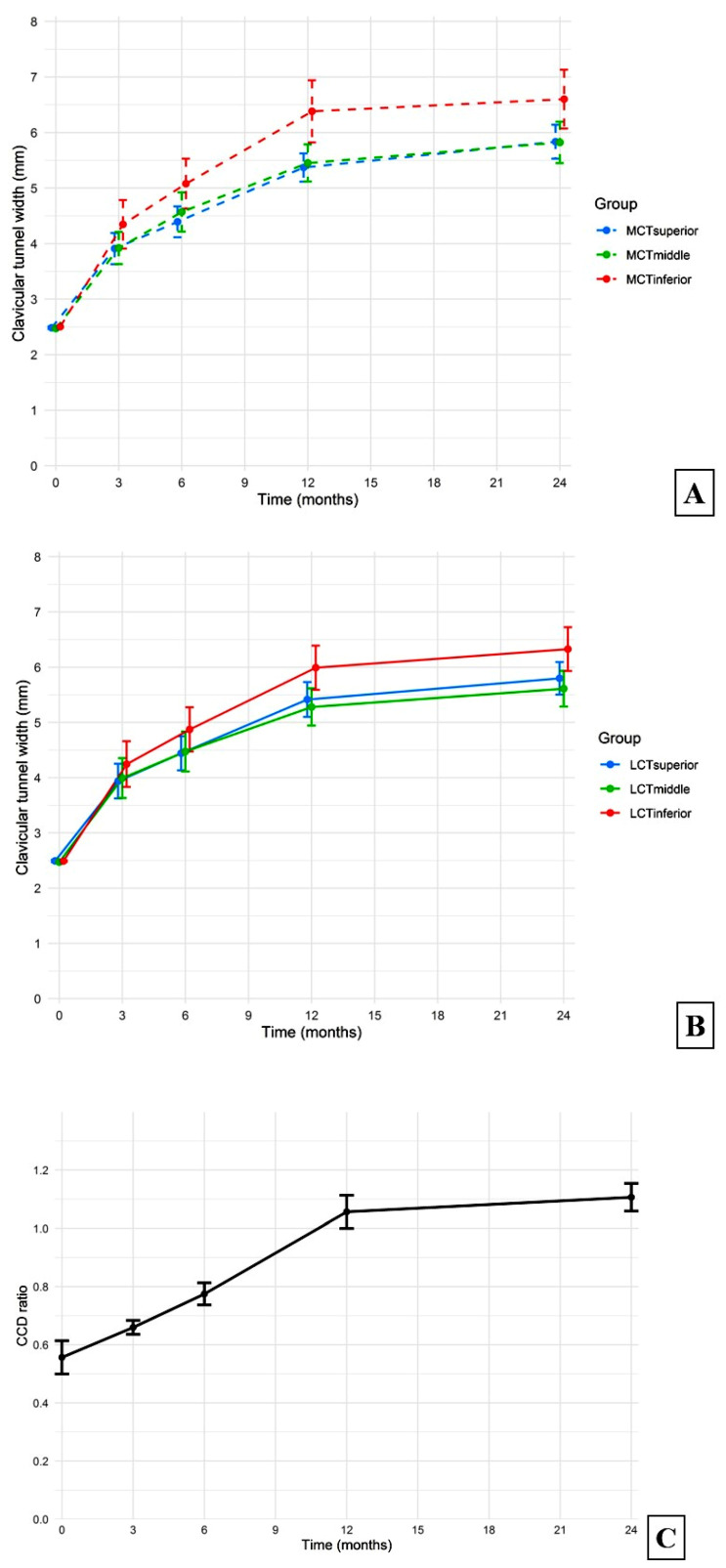
Radiographic follow-ups of mean and standard deviations of the (**A**) medial and (**B**) lateral clavicular tunnel widths and (**C**) coracoclavicular distance (CCD) ratios at different time points.

**Figure 5:**
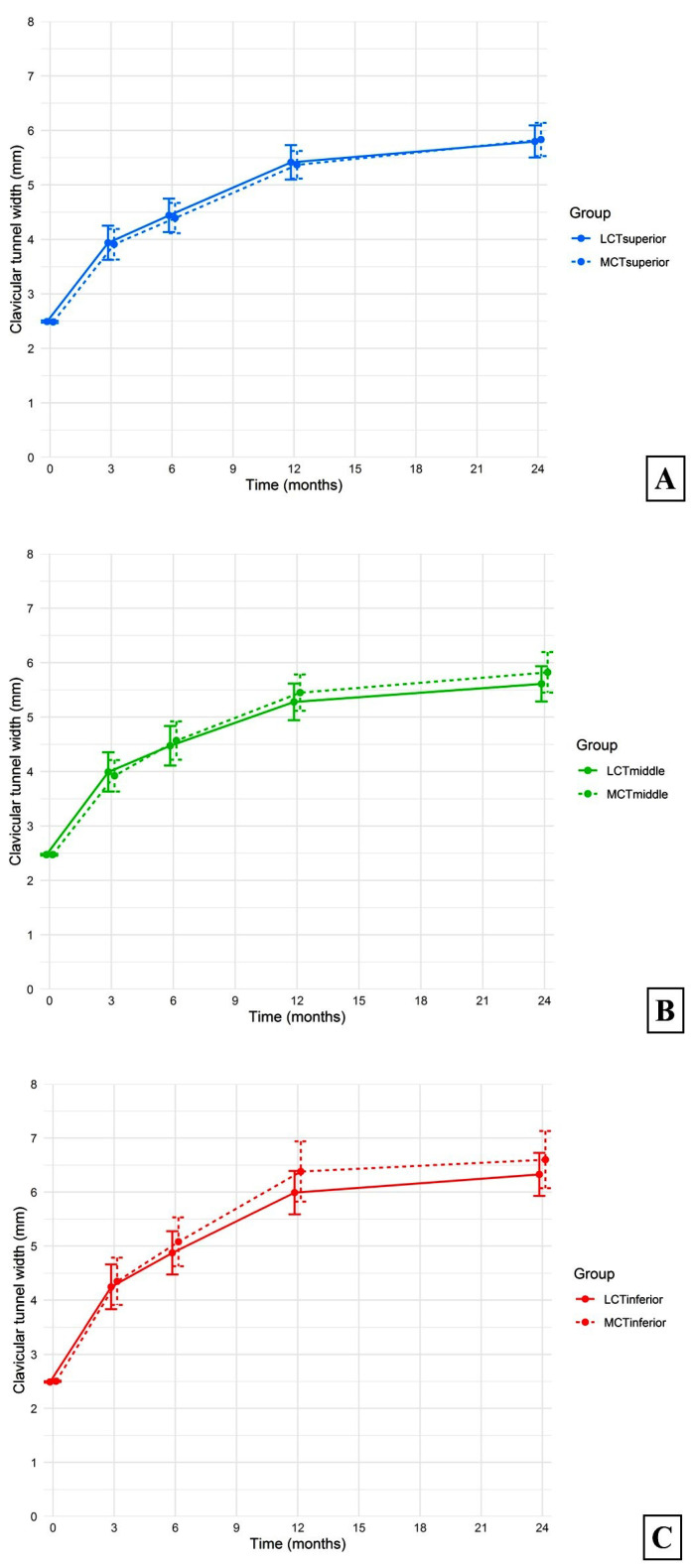
The average medial and lateral clavicular tunnel widths at the (**A**) superior, (**B**) middle, and (**C**) inferior levels.

At all regular follow-up periods, significant increases in the average widths of the medial and lateral clavicular tunnels were found compared with the immediate postoperative measurements (all *p*-values < 0.05) (Figure 4A,B). There were no significant differences in mean superior, middle, or inferior levels of clavicular tunnel widths between the medial and lateral clavicular tunnels at all time points (Figure 5A–C). The *p*-values of the comparisons between the medial and lateral clavicular tunnels at the 3-month, 6-month, 12-month, and 24-month follow-ups at the superior level were 0.92, 0.83, 0.85, and 0.82, respectively, at the middle level 0.75, 0.68, 0.44, and 0.36, respectively, and at the inferior level 0.75, 0.50, 0.24, and 0.40, respectively.

The radiographic CCD ratios immediately postoperatively and at the routine follow-ups are shown in Figure 4C. There were significant correlations between widening of both medial and lateral clavicular tunnels at the superior, middle, and inferior levels and increases in the CCD, as shown in Table 1, but there were no correlations between age, sex, or time of surgery and increases in the CCD at all follow-up points (all *p*-values > 0.05). The average ASES score at the 24-month follow-ups was 95.28 ± 3.62, and the ASES scores were not significantly different between the adequate reduction (N = 16) and loss of reduction (N = 7) groups (*p*-value = 0.608).

Two patients had developed osteolysis of the distal clavicle at the 12-month follow-ups, with one further patient at the 24-month follow-ups; five patients had developed CC ossification at the 12-month follow-ups, with no further incidences at the 24-month follow-ups; two patients had developed AC joint arthritis at the 12-month follow-ups, with one further patient at the 24-month follow-ups. At the 24-month follow-ups, the ASES scores were not significantly different between the patients with and without CC ossification (*p*-value = 0.192) or between the patients with and without AC joint arthritis (*p*-value = 0.533).

Power calculations were performed to assess the adequacy of our sample size for detecting significant correlations between the clavicular tunnel widths (medial and lateral) at the superior, middle, and inferior levels and the CCD ratios. The analysis revealed high powers for detecting significant correlations: the power for the medial clavicular tunnel widths was 0.995 at the superior level, 0.992 at the middle level, and 0.997 at the inferior level. Similarly, the power for the lateral clavicular tunnel widths was 0.995 at the superior level, 0.995 at the middle level, and 0.989 at the inferior level. The intra-observer and inter-observer agreements ranged from 0.985 to 0.995. 

### 3.2. Complications

There were no reports of any postoperative clavicular fractures or surgical wound infections at the 24-month follow-ups.

## 4. Discussion

Our study found that a significant number of patients had widening of both medial and lateral coracoid tunnels after CC stabilization and AC capsule repair with loss of reduction, and a significant correlation between the degree of tunnel widening and radiographic loss of reduction, although the functional outcomes were not affected. 

Various previous studies have reported on the impact of clavicular tunnel widening after CC stabilization. Thangaraju et al. [12] reported significant clavicular tunnel widening after arthroscopic-assisted CC stabilization using a single-tunnel button–tape construct with AC joint tape cerclage, but they found no correlation between clavicular tunnel widening and loss of reduction at a median follow up period of 4.5 months. However, Cook et al. [16] reported postoperative loss of reduction in eight of ten patients after 6-month postoperative follow-ups for CC stabilization with GraftRope (Arthrex Inc., Naples, FL, USA), and tunnel widening was noted in all of their patients. 

A difference in progressive clavicular tunnel widening between CC stabilization and reconstruction was evident in recent studies. One study demonstrated significant increases in tunnel sizes during the early postoperative period, noting a median tunnel size change from 3 mm immediately postoperative to 5 mm at a later follow-up, a 66.6% increase [12]. This was consistent across both medial and lateral clavicular tunnels at the superior, middle, and inferior levels. Another investigation also found significant tunnel widening with different acromioclavicular joint repair devices, showing a notable increase in both the superior clavicular and inferior coracoid regions [17]. These and other studies indicate that tunnel widening is a common occurrence across various stabilization devices, potentially contributing to a higher fracture risk, particularly in high-impact athletes. This needs to be carefully considered in the surgical planning and postoperative management to mitigate long-term complications associated with tunnel widening. In our study, the mean clavicular tunnel widths had increased at the 24-month postoperative follow-ups by approximately 140% at both the medial and lateral tunnels (initial diameter of 2.5 mm to an average increase to 6 mm) compared with the mean clavicular tunnel widths immediately postoperatively. We hypothesize that the observed increases in tunnel sizes may be attributed to substantial micromotion between the suture material and the bone, which likely resulted in tunnel widening due to the suture material slowly wearing down the bone. Although significant widening of the clavicular tunnels was observed in our study, there were no patients with postoperative clavicular fractures as reported in previous studies [10,18,19,20]. However, we found statistically significant correlations between the widening of the clavicular tunnels and postoperative loss of reduction, with increased clavicular tunnel widths observed during the 2-year follow-ups, even though the ligaments had completely healed. The slope of tunnel widening decreased after 6–12 months. A possible cause of accelerated tunnel widening and superior migration of the clavicle in the first period was the micromotion of the suture material, which was the primary stabilizer during that period. Then, as the ligament fully healed, the superior migration of the clavicle still continued until the healed ligament was tight, which then acted as the primary stabilizer instead of the suture. The basic goal of CC stabilization in acute high-grade AC joint injury is to induce healing of the CC ligament by reducing and maintaining the CC interspace [21]. Therefore, we recommend that all hardware and suture materials used in this procedure should be removed as soon as possible following full ligament healing. Early removal of these materials can help to minimize the mechanical stress and potential damage, thus allowing the healed CC ligament to maintain its stabilizing role effectively without the interference of suture movement. This approach is particularly important even if the CC distance indicates that healing is not yet fully realized, as it prevents long-term consequences that could jeopardize the success of the surgery.

There are other factors besides tunnel widening which can lead to loss of reduction following CC stabilization. Seo et al. [8] reported that the reduction-first technique between the coracoid tunnel and clavicular tunnel gave significantly better clinical and radiological outcomes than the tunnel-first technique in single-tunnel CC stabilization. They hypothesized that deviations of the clavicular tunnel angle may increase tunnel wall stress, engendering tunnel widening. Gu et al. [22] compared the treatment of acute AC joint dislocation between single and double TightRope systems and they found the double TightRope system was more reliable for reduction maintenance when compared with the single TightRope system, but there were no differences in clinical outcomes. To avoid postoperative loss of reduction, the surgeon should decrease the opportunity for postoperative windscreen wiper micromotion of the suture material in the bone tunnel. We recommend creating two clavicular tunnels with the smallest size holes practical and using the coracoid-based landmarks technique; a reduction in the AC joint before creating the clavicular tunnels; and removing the sutures as soon as full and stable CC ligament and AC capsule healing are confirmed.

Postoperative loss of reduction, commonly occurring after CC stabilization and AC capsule repair, is a significant concern as it may influence the functional outcomes of the surgery. Earlier studies have found that while the loss of anatomical reduction did not always affect the basic shoulder functions, as measured by functional scores, it could significantly impact more specialized outcomes. For instance, Çarkçı and team (2020) observed that a reduction loss of more than 3 mm did not affect the Constant scores significantly, but did lead to statistically significant impairments as assessed by Acromioclavicular Joint Instability scores, subjective evaluations, and the aesthetic satisfaction of the patients [23]. Furthermore, Maziak and team (2019) suggested that radiological outcomes, such as dynamic posterior translation, could be predicted by the quality of the initial reduction and the stabilization technique used [24]. They reported that poor radiological outcomes did not necessarily correlate with poor clinical outcomes, suggesting that some patients could maintain function despite radiological evidence of instability. Thus, the impact of postoperative loss of reduction on functional outcomes is nuanced. It appears that while basic shoulder function may remain largely unaffected, specific aspects related to the stability of the joint and aesthetic concerns, which contribute to the overall satisfaction and perceived recovery quality, can be significantly compromised.

The incidence of postoperative CC ossification and AC joint arthritis in combined CC stabilization and AC capsule repairs were also examined in our study, which found similar incidences as previous studies. Choi et al. [25]. reported midterm results of CC stabilization with double augmentation and they found 27.9% of their patients, compared with 21% in our study, had developed postoperative ossification of the CC ligament. They hypothesized that CC ossification occurred when bone marrow cells migrated along a torn CC ligament and passed through the clavicular tunnels that had been drilled in the bone. There has been no evidence that CC ossification had or has any adverse effects on clinical outcomes, as further shown by the study of Ertogurul and team [26]. Our study also found no significant differences in clinical outcomes between patients with and without CC ossification. Shin et al. [27]. studied complications after arthroscopic CC stabilization using a loop suspensory fixation device and they found 5.5% of their patients (13% in our study) developed AC joint arthritis. As in our study, they detected radiographic AC joint arthritis as early as 6 months postoperatively. This result may be similarly understood in terms of joint dislocation in other joints or age-related factors. A high-grade AC joint injury and subsequent CC stabilization and AC capsule repair procedure significantly alter the environment of the AC joint, and despite surgical reduction, there may be some future degeneration. However, in our study, no clinical symptoms appeared in any of our patients during the 24-month follow-up period.

There were several limitations to this study. First, all radiographs were reviewed retrospectively, therefore the quality of the radiographs could not be controlled. Second, all radiographic parameters were evaluated in only one dimension, and further investigations using computed tomography (CT) would provide more accuracy for detecting and calculating clavicular tunnel widths and CCDs. Third, our surgical technique for this procedure during the study period used the overreduction technique of intraoperatively decreasing the CCD to approximately 50% of the unaffected side, and studies based on the standard anatomical reduction technique may have different results.

## 5. Conclusions

Postoperative widening of the clavicular tunnels was correlated with postoperative loss of reduction. To minimize the effects of this problem we recommend that the hardware and suture material used during this procedure should be removed as soon as full healing of the ligament is confirmed to minimize tunnel widening and reduce future widening. This strategy aims to minimize tunnel widening and prevent its progression, thereby improving the surgical outcomes.

## Figures and Tables

**Table 1:** The correlations between the widening of both medial and lateral clavicular tunnels and the increased coracoclavicular distance (CCD) ratios.

Variable Correlation with Increasing of the CCD Ratio	Spearman’s Rho Correlation Coefficient	*p*-Value
MCTsup	0.764	<0.001
MCTmid	0.762	<0.001
MCTinf	0.732	<0.001
LCTsup	0.761	<0.001
LCTmid	0.743	<0.001
LCTinf	0.773	<0.001

MCTsup, superior level of the medial clavicular tunnel; MCTmid, middle level of the medial clavicular tunnel; MCTinf, inferior level of the medial clavicular tunnel; LCTsup, superior level of the lateral clavicular tunnel; LCTmid, middle level of the lateral clavicular tunnel; LCTinf, inferior level of the lateral clavicular tunnel.

## Data Availability

The data used to support the findings of this study are available from the corresponding author upon request.
